# Parkinson's Disease and Home Healthcare Use and Expenditures among Elderly Medicare Beneficiaries

**DOI:** 10.1155/2015/606810

**Published:** 2015-05-24

**Authors:** Sandipan Bhattacharjee, Aaron Metzger, Cindy Tworek, Wenhui Wei, Xiaoyun Pan, Usha Sambamoorthi

**Affiliations:** ^1^Department of Pharmacy Practice and Science, School of Pharmacy, The University of Arizona, Tucson, AZ 85721, USA; ^2^Department of Life-Span Developmental Psychology, West Virginia University, Morgantown, WV 26506, USA; ^3^Department of Pharmaceutical Systems & Policy, School of Pharmacy, West Virginia University, Morgantown, WV 26506, USA; ^4^Sanofi US, Bridgewater, NJ 08807, USA; ^5^Evidera, Lexington, MA 02420, USA

## Abstract

This study estimated excess home healthcare use and expenditures among elderly Medicare beneficiaries (age ≥ 65 years) with Parkinson's disease (PD) compared to those without PD and analyzed the extent to which predisposing, enabling, need factors, personal health choice, and external environment contribute to the excess home healthcare use and expenditures among individuals with PD. A retrospective, observational, cohort study design using Medicare 5% sample claims for years 2006-2007 was used for this study. Logistic regressions and Ordinary Least Squares regressions were used to assess the association of PD with home health use and expenditures, respectively. Postregression nonlinear and linear decomposition techniques were used to understand the extent to which differences in home healthcare use and expenditures among elderly Medicare beneficiaries with and without PD can be explained by individual-level factors. Elderly Medicare beneficiaries with PD had higher home health use and expenditures compared to those without PD. 27.5% and 18% of the gap in home health use and expenditures, respectively, were explained by differences in characteristics between the PD and no PD groups. A large portion of the differences in home healthcare use and expenditures remained unexplained.

## 1. Introduction

Elderly individuals with PD experience substantially higher healthcare expenditures compared to those without PD [1–4]. Specifically, home healthcare expenditures have been observed to be consistently and substantially higher among elderly individuals with PD compared to those without PD [1–4]. The magnitude of the difference in home healthcare expenditure varies depending on study population as well as the year in which the studies were conducted. Existing literature suggests that in the United States (US) the average annual home healthcare expenditures among elderly individuals with PD can be as high as 3.2 times more compared to those without PD [2]. Home healthcare use has also been shown to be higher among elderly individuals with PD compared to those without PD. Rates of home healthcare use have been observed to vary from twofold [1, 3] to more than threefold [2] higher among elderly individuals with PD compared to those without PD in the USA.

As PD has higher prevalence among elderly individuals (≥65 years) than among other age groups, most of the elderly living in the United States (US) are eligible for Medicare. Therefore, most of home healthcare spending will be borne by Medicare. It has been estimated that total Medicare home healthcare expenditures increased from $8.5 billion in 2000 to $18 billion in 2012, a 112% increase [5]. Because of the accelerated growth of home healthcare expenditures the Centers for Medicare and Medicaid Services (CMS) agency has been exploring ways to reduce the excess home healthcare expenditures. Thus, an understanding of factors contributing to home healthcare among all the elderly and specifically those with PD who are high utilizers of home healthcare is critical. However, to the best of authors' knowledge, no study has analyzed various factors associated with higher home healthcare use and expenditures among individuals with PD. Therefore, the aims of this study were to (i) estimate excess home healthcare use and expenditures associated with PD, (ii) examine factors associated with excess home healthcare use and expenditures, and (iii) quantify the extent to which each of the different sets of factors explains excess home healthcare use and expenditures among individuals with PD compared to those without PD.

## 2. Methods

### 2.1. Conceptual Framework

The conceptual behavioral model by Andersen was used to examine the predisposing, enabling, need, personal health choices, and external environmental factors associated with the healthcare expenditures [6]. The Andersen Behavioral Model (ABM) has been used in various studies related to usage of health services (both healthcare use and expenditures). The ABM posits an individual's use of health services as a function of (1) predisposing, (2) enabling, (3) need factors, (4) personal health choices, and (5) external environment. The individual's characteristics that are predisposing include demographic characteristics (e.g., gender, age, and race/ethnicity). The ability of an individual to access a health service is termed as an enabling factor (e.g., public assistance). Need factors are represented either by a subjective acknowledgement of need such as a patient's symptoms or by a professional's judgment of the need for healthcare based on disease characteristics (e.g., number of comorbidities). Personal health choices (e.g., substance use) and external environment characteristics (e.g., census region, metro status) also influence an individual's use of health services.

#### 2.1.1. Study Design

We used a retrospective, observational, cohort study design with 12-month baseline and 12-month follow-up period. For the purposes of this study, Medicare 5% sample claims database for years 2006-2007 was used. Calendar year 2006 was considered as the baseline period and calendar year 2007 was considered as the follow-up period.

#### 2.1.2. Data Source

The data were derived from the Medicare 5% sample, which contains all final action claims data for a random 5% sample of all claims of Medicare beneficiaries [7]. Available standard analytic files (SAF) in Medicare 5% sample claims database are (1) inpatient, (2) outpatient (encompassing claims from physician office), (3) skilled nursing facility, (4) carrier, (5) hospice care, (6) home health, and (7) durable medical equipment analytic data files. The Medicare 5% sample claims database can be used to conduct both cross-sectional and longitudinal studies. A unique, deidentified Medicare beneficiary identifier is assigned to each enrollee, which is used to follow them longitudinally. Several studies have been conducted using this database [8–10]. The information recorded in the claims dataset includes dates of service provided, charge and payment amounts, clinical diagnosis codes, and procedure codes. Information on demographic characteristics such as age, gender, and race/ethnicity is available in the denominator files of Medicare claims dataset.

The home healthcare files consist of fee-for-service claims for Medicare-certified home health services, service dates, claim payment amount, primary diagnosis code (ICD-9-CM codes), up to 9 other diagnosis codes, and the total number of home healthcare visits.

**Figure 1:**
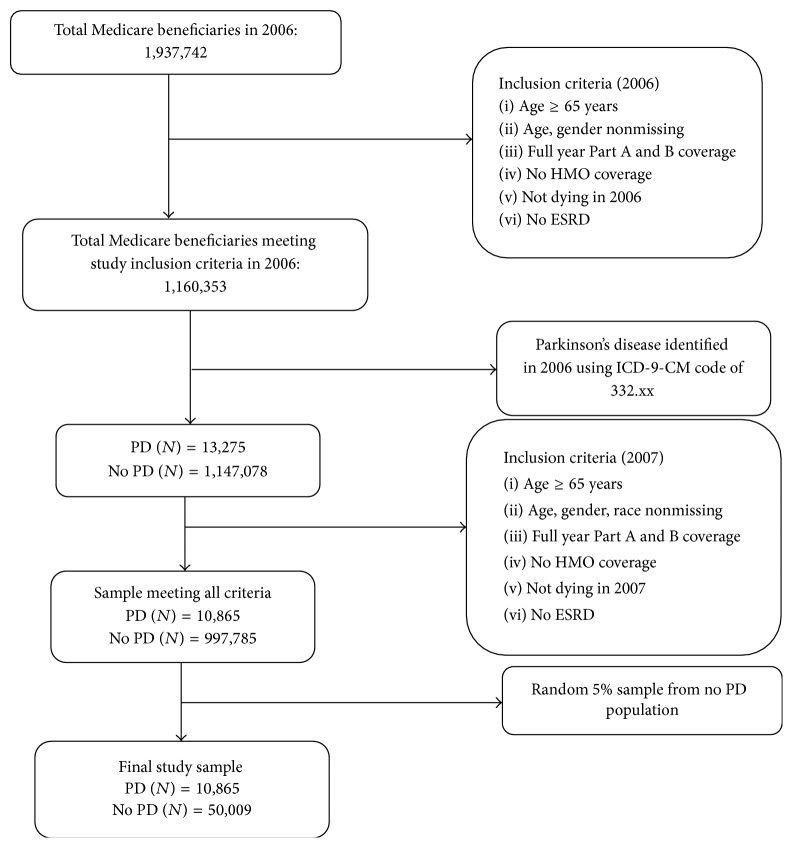
Cohort development.

*Study Sample*. Our study sample comprised community-dwelling elderly Medicare beneficiaries aged 65 years and older, who were alive and had continuous Part A and Part B enrollment during 2006 and 2007. Medicare beneficiaries enrolled in Health Maintenance Organizations (HMO) were excluded due to unavailability of HMO claims. To be included in the final study sample, individuals with and without PD were required to have positive direct healthcare expenditures. Individuals with end-stage renal disease (ESRD) were also excluded from the final sample. The final sample size was 60,874 with 10,865 elderly Medicare beneficiaries with PD and 50,009 elderly Medicare beneficiaries without PD. The cohort development of this study is shown in Figure 1.

#### 2.1.3. Measures

*Dependent Variable*

*Home Healthcare Use*. Home healthcare use was identified during the follow-up period (i.e., calendar year 2007). Elderly Medicare beneficiaries with any paid home healthcare visit during the follow-up period were considered to be home health users.

*Home Healthcare Expenditures*. Among users of home healthcare, we examined home healthcare expenditures during follow-up period (i.e., calendar year 2007). We used actual Medicare payments to calculate home healthcare expenditures from home healthcare file. Home health expenditure was skewed to the right and, therefore, we transformed expenditures with natural logarithm.

### 2.2. Key Independent Variable: PD and No PD

Presence or absence of PD constituted the key independent variable for this study and this was measured during baseline period. Individuals with PD were identified using International Classification of Diseases, Ninth Revision, Clinical Modification (ICD-9-CM) codes of 332.xx. Use of ICD-9-CM codes from Medicare claims to identify PD has modest sensitivity (61.13%) and positive predictive value (65.13%) and the very high specificity (99.08%) [11].

### 2.3. Independent Variables

All independent variables were measured during the baseline period. The* predisposing* characteristics comprised gender (women/men), race/ethnicity (white, African American, Latino, and others), and age (65–74 and 75 years and older).* Enabling* characteristics consisted of public assistance (which was indicated by Medicare premiums and deductibles that were subsidized by the state keeping in view the financial status of the enrollee). The* need factors* comprised the number of other cooccurring comorbidities.* Personal health choices* consisted of substance use disorders that included alcohol, tobacco, and drug use. This domain also included baseline healthcare use. The* external environment* factors comprised census region and metro status.

#### 2.3.1. Statistical Methods

Statistically significant differences in home healthcare use during follow-up period by presence of PD were ascertained with chi-square tests. Logistic regressions were used to examine the association between home healthcare use and PD after controlling for predisposing, enabling, need factors, personal health choices, and external environment.

We examined the differences in average home healthcare expenditures between PD and no PD groups by using *t*-tests. We also conducted Ordinary Least Squares (OLS) regressions on log-transformed home healthcare expenditures during follow-up period to examine the relationship between PD and home healthcare expenditures after controlling for predisposing, enabling, need factors, personal health choices, and external environment. The relationship between home healthcare use and expenditures for each characteristic was ascertained by using chi-square tests and *t*-tests for every characteristic between PD and no PD groups.

*Postregression Decomposition Techniques*. To assess the extent to which differences in home healthcare use and expenditures among individuals with and without PD can be explained by predisposing, enabling, need factors, personal health choices, and external environmental factors, we used postregression decomposition techniques. The differences in home healthcare use or expenditures between the two groups of individuals with and without PD are compartmentalized into two parts: (1) one part is attributable to the differences in characteristics, which is the explained portion of the differences, and (2) the other part is the differences attributable to differences in coefficients. The explained portion of the gap is the sum of the differences between individuals with and without PD in terms of the observed or measured characteristics weighted by the estimated coefficients of individuals with PD.

For home healthcare expenditures, we used postregression linear decomposition [12, 13]. For home healthcare use, a postregression nonlinear decomposition was used because home healthcare use was measured as a binary variable [14]. In the nonlinear decomposition, the contribution of each of the individual-level factors to the gap in terms of home healthcare use between the two groups is estimated by the change in the mean predicted probability by superimposing the distribution of elderly Medicare beneficiaries with PD on those without PD, keeping all other factors constant [14].

A common problem encountered with decomposition techniques is the “index number” problem. As the characteristics can be weighted by the regression coefficients from PD group or no PD group, the estimates of explained portion will vary. Many solutions have been proposed to overcome this problem [15–18]. For the current study we used the solution proposed by Neumark and used the estimated regression coefficients from a regression combining PD and no PD group in which presence of PD was not used as an independent variable [15].

Analyses were conducted using SAS version 9.3 (SAS Institute Inc., Cary, NC). STATA version 13.0 was used to perform nonlinear decomposition, whereas Blinder-Oaxaca linear decomposition was performed using Microsoft Excel 2013.

## 3. Results

Table 1 describes the study sample overall as well as by PD status. The final study sample consisted of 60,874 elderly Medicare beneficiaries among whom 10,865 had PD and 50,009 did not have PD. Overall, the majority of the study participants comprised women (58.3%) and whites (89.8%) and were 75 years and older (51.2%). The average number of comorbidities in the overall study sample was 17.26 (±10.38). In terms of predisposing, enabling, need, personal health choice, and external environmental factors, all the characteristics were statistically significantly different between elderly Medicare beneficiaries with and without PD except for baseline substance use disorder and cancer. Among elderly Medicare beneficiaries with PD, a higher percentage were men (50.1%) and among those without PD a significantly lower percentage were men (39.9%). A greater proportion of elderly Medicare beneficiaries with PD were 75 years and older (63.9%) and a lower proportion of elderly Medicare beneficiaries without PD were 75 years and older (48.4%). The study sample consisted of an overwhelming majority of whites in both the groups (around 90% in both groups). A higher percentage of elderly Medicare beneficiaries were on public assistance among those with PD (16.0%) compared to those without PD (11.4%). Elderly Medicare beneficiaries with PD (21.97 ± 11.75) had a significantly higher number of comorbidities compared to those without PD (16.24 ± 9.76). Elderly Medicare beneficiaries with PD had significantly higher rates of baseline inpatient, outpatient, hospice, skilled nursing facilities, and durable medical equipment use. An overwhelmingly higher percentage of elderly Medicare beneficiaries resided in the metro regions in both the two groups (approximately 80% in both groups). Elderly Medicare beneficiaries in both PD and no PD groups resided primarily in the Southern census region (around 38% in both groups) followed by Midwest, Northeastern, and Western regions.

Table 2 exhibits the relationship between predisposing, enabling, personal health choice, need, and external environmental factors and home healthcare use and average home healthcare expenditure by PD status. There were 2,445 and 4,061 elderly Medicare beneficiaries with and without PD, respectively, who had home healthcare use. Overall, 22.5% of the elderly with PD used home healthcare compared to 9.2% without PD (*p* < .001). This translated into a 13.3-percentage-point difference in home healthcare use. For every characteristic (except for elderly Medicare beneficiaries with skilled nursing facility visits), home healthcare use was higher among the elderly with PD compared to those without PD (Table 2). For example, the home healthcare use among women (24.9% versus 10.5%) and men (20.2% versus 7.3%) with PD was significantly higher compared to those without PD. Table 2 also summarizes the average home healthcare expenditures among home healthcare users by the presence or absence of PD. The average home healthcare expenditures among elderly Medicare beneficiaries (who used home healthcare service) with PD were 1.37 times higher compared to those without PD ($6,792 versus $4,965). Home healthcare expenditures were also significantly higher across each characteristic among the elderly with PD compared to those without PD. For example, average home healthcare expenditures among women ($7,055 versus $5,201) and men ($6,469 versus $4,453) with PD were significantly higher compared to those without PD.

Table 3 summarizes the findings from the logistic regression analyses of home healthcare use and OLS regressions analyses on log-transformed home healthcare expenditures on a pooled sample among elderly Medicare beneficiaries who had used home healthcare. It was observed that, after controlling for predisposing, enabling, need, personal health choice, and external environmental factors, elderly individuals with PD were more than two times (Adjusted Odds Ratio: 2.12; 95% Confidence Interval: 1.99–2.25) as likely to have home healthcare use as those without PD. It can be noted from the OLS regression analyses that elderly Medicare beneficiaries with PD had 39% (calculated using the formula exp^(*β*)^ − 1) higher home healthcare expenditure compared to those without PD.

Table 4 summarizes the results from the nonlinear and linear decomposition on the home healthcare use (yes/no) and expenditures. There was a 13.3-percentage-point difference in home healthcare use between these two groups in terms of home healthcare use. From the multivariate decomposition analysis, it was observed that, out of the 13.3-percentage-point difference in home healthcare use among elderly Medicare beneficiaries, a 3.64-percentage-point difference was explained by the individual-level variables included in this study. Thus, a 27.5% difference in home healthcare use among elderly Medicare beneficiaries with and without PD was explained by the predisposing, enabling, need, personal health choice, and external environmental factors. Need characteristics (physical and mental health conditions and baseline resource use) of the elderly Medicare beneficiaries explained the highest (approximately 65%) proportion of the home healthcare use differences between elderly Medicare beneficiaries with and without PD.

Table 4 also displays the results of linear decomposition of home healthcare expenditures among elderly Medicare beneficiaries with and without PD. We restricted these analyses to users of home healthcare. The mean log-transformed home healthcare expenditure among elderly Medicare beneficiaries with and without PD was 8.44 and 8.09 units, respectively. This translated into a 0.35-unit difference in mean log-transformed home healthcare expenditure. Using pooled regression coefficients, 18% of the home healthcare expenditures differences among elderly Medicare beneficiaries with and without PD were explained, while the remaining majority (82%) of the home healthcare expenditure differences remained unexplained. It was observed that baseline personal health choices explained the highest proportion of the home healthcare expenditure differences followed by need factors. Using the pooled weights, it can be inferred that if the baseline personal health choices were similar between elderly Medicare beneficiaries with and without PD, then the home healthcare expenditure would decrease by 12.6%. The negative coefficient of the enabling characteristic signifies that if the gender, race/ethnicity, and age characteristics of elderly Medicare beneficiaries with and without PD were similar, then the difference in home healthcare use would increase by the respective percentage units.

## 4. Discussion

The present study examined the magnitude of the difference in home healthcare resource use and expenditures among elderly Medicare beneficiaries with and without PD. This study also evaluated the extent to which predisposing, enabling, need, personal health choices, and external environmental factors explained the differences among elderly Medicare beneficiaries with and without PD. It was observed that, after adjusting for the predisposing, need, personal health choice, and external environmental factors, elderly Medicare beneficiaries with PD had 13.3% higher home healthcare use and 39% higher home healthcare expenditures (among users) compared to those without PD. Previous studies have consistently found that home healthcare use and expenditure among individuals with PD are significantly higher among individuals with PD [1–4]. However, no study examined the factors associated with higher home healthcare use and expenditures among elderly individuals with PD and to what extent the individual-level factors explain such differences. The unique contribution of this study is providing an assessment of the extent to which these individual-level factors explained the use and expenditure differences using a nationally representative sample of Medicare beneficiaries.

Approximately 27% and 18% of the home healthcare use and expenditure differences among elderly Medicare beneficiaries with and without PD were explained by the individual-level factors adjusted in this study. The need characteristics such as baseline comorbidities and personal health choices such as baseline resource use explained the highest proportion of the explained differences. The high unexplained portions for home healthcare use (72.5%) and expenditure (82%) difference between elderly Medicare beneficiaries with and without PD can be due to several factors such as severity and duration of PD, medication adherence, and inclination towards seeking healthcare. Moreover, the presence or absence of a caregiver for elderly Medicare beneficiaries with PD can also be a contributing factor to the difference.

Findings from this study have profound implications for healthcare providers, payers, and patients. Findings from the decomposition analysis identified the factors that contribute to the excess burden of home healthcare among elderly Medicare beneficiaries with PD. The study findings can be useful to the Centers for Medicare and Medicaid Services (CMS) in formulating programs and policies that can help reduce home healthcare use and expenditures among elderly Medicare beneficiaries with PD, thereby having a potential to bend the ever increasing Medicare cost curve. For example, the decomposition analyses in this study showed that one of the major drivers of home healthcare expenditure is the presence of baseline comorbidities. CMS agency is currently implementing reimbursement policies that can encourage management of Medicare beneficiaries with multiple chronic conditions. The study findings are important for providers as well as suggesting a need for collaborative care among different specialists such as neurologists, endocrinologists, and mental health specialists to provide a complete and holistic care to these elderly individuals with chronic diseases. Our study findings reinforce the implementation of an integrated delivery system (IDS) where primary physicians serve as the gate-keeper and maintain a good referral system, which can also be helpful in treating elderly individuals with multiple chronic conditions. In light of the recent Affordable Care Act (ACA), there is emphasis on team-based approaches such as Patient Centered Medical Home (PCMH) where continuous and well-coordinated care is provided by a team of healthcare providers [19]. The aims of these PCMHs are to provide evidence based treatments for acute and chronic conditions, as well as providing preventive services. These types of care models can be beneficial to the elderly patients as they have the potential to improve health outcomes, which in turn can reduce service use and expenditures. Such models can also reduce the out-of-pocket spending for the patients while improving their health outcomes.

The study strengths are large sample size, representative sample, and comprehensive list of variables. While interpreting the findings the following limitations need to be noted. The findings from this study are not generalizable to other populations or settings. HMO enrollees are excluded from the study sample. Users of home healthcare may be different from nonusers of home healthcare in unobserved variables. Our study does not control for such selection bias. Five percent Medicare sample did include many variables that are associated with home healthcare use. This may lead to underestimation of the explained portion of the estimated differences in home healthcare expenditures among individuals with and without PD. However, by using a representative sample of Medicare beneficiaries with linked Medicare claims, we were able to estimate the extent to which unmeasured factors can underestimate explained portion of the differences in expenditures among individuals with and without PD.

## 5. Conclusion

Notwithstanding the limitations, this study examined the magnitude of the difference in home healthcare resource use and expenditure among elderly Medicare beneficiaries with and without PD and also evaluated the extent to which predisposing, enabling, need, personal health choice, and external environmental factors explained these differences. Elderly individuals with PD had 13.3% and 39% higher home healthcare use and expenditure compared to those without PD. Individual-level factors used in this study explained 27.5% and 18% of the differences in home healthcare use and expenditures, respectively. Future studies should compare the home healthcare use and expenditure among elderly individuals with PD compared to other neurodegenerative diseases (e.g., Alzheimer's disease) and include some of the factors such as functional status, informal caregiving, severity of chronic conditions, and medication use, to understand their influence on the home healthcare use and expenditures.

## Figures and Tables

**Table 1:** Description of study sample, overall and by Parkinson's disease status among Medicare beneficiaries. National Medicare 5% sample, 2006-2007.

Variables	Overall	Col.%	PD	Col.%	No PD	Col.%	Sig.
	*N* = 60,874		*N* = 10,865		*N* = 50,009		
Predisposing characteristics							
Gender							*∗∗∗*
Female	35,470	58.3	5,421	49.9	30,049	60.1	
Male	25,404	41.7	5,444	50.1	19,960	39.9	
Race/ethnicity							*∗∗∗*
White	54,652	89.8	10,007	92.1	44,645	89.3	
AA	3,695	6.1	425	3.9	3,270	6.5	
Others	2,527	4.2	433	4.0	2,094	4.2	
Age group							*∗∗∗*
65–74	29,725	48.8	3,927	36.1	25,798	51.6	
75, +	31,149	51.2	6,938	63.9	24,211	48.4	

Enabling characteristics							
Public assistance							*∗∗∗*
Yes	7,445	12.2	1,742	16.0	5,703	11.4	
No	53,429	87.8	9,123	84.0	44,306	88.6	

External environmental characteristics							
Census region							*∗∗∗*
Northeastern	11,983	19.7	2,399	22.1	9,584	19.2	
Midwest	15,842	26.0	2,837	26.1	13,005	26.0	
South	23,323	38.3	3,931	36.2	19,392	38.8	
West	9,726	16.0	1,698	15.6	8,028	16.1	
Metro status							*∗∗∗*
Metro	46,162	75.8	8,448	77.8	37,714	75.4	
No metro	14,712	24.2	2,417	22.2	12,295	24.6	

Personal health practices							
Substance use							
Yes	2,337	3.8	440	4.0	1,897	3.8	
No	58,537	96.2	10,425	96.0	48,112	96.2	
Inpatient visit							*∗∗∗*
Yes	12,875	21.2	3,584	33.0	9,291	18.6	
No	47,999	78.8	7,281	67.0	40,718	81.4	
DME visit							*∗∗∗*
Yes	19,239	31.6	4,679	43.1	14,560	29.1	
No	41,635	68.4	6,186	56.9	35,449	70.9	
Hospice visit							*∗∗∗*
Yes	256	0.4	122	1.1	134	0.3	
No	60,618	99.6	10,743	98.9	49,875	99.7	
SNF visit							*∗∗∗*
Yes	3,576	5.9	1,597	14.7	1,979	4.0	
No	57,298	94.1	9,268	85.3	48,030	96.0	
Outpatient visit quintile							*∗∗∗*
0-0	14,403	23.7	1,708	15.7	12,695	25.4	
1-1	10,153	16.7	1,469	13.5	8,684	17.4	
2-3	13,303	21.9	2,306	21.2	10,997	22.0	
4–6	10,654	17.5	2,234	20.6	8,420	16.8	
7–178	12,361	20.3	3,148	29.0	9,213	18.4	
Office visit quintile							*∗∗∗*
0–5	9,433	15.5	502	4.6	8,931	17.9	
6–10	12,697	20.9	1,335	12.3	11,362	22.7	
11–16	12,676	20.8	2,082	19.2	10,594	21.2	
17–25	12,058	19.8	2,632	24.2	9,426	18.8	
26–281	14,010	23.0	4,314	39.7	9,696	19.4	

Need factor							
	Average	S.D.	Average	S.D.	Average	S.D.	
Number of comorbidities	17.26	10.38	21.97	11.75	16.24	9.76	*∗∗∗*

Note: based on 10,865 and 50,009 elderly (age 65 or older) Medicare beneficiaries with and without Parkinson's disease who were continuously enrolled in Medicare Part A and Part B during 2006 and 2007 and were not enrolled in Health Maintenance Organizations during 2006 and 2007; they had a positive direct total healthcare expenditure; individuals with end-stage renal disease (ESRD) as well as those without full year enrollments due to death or some other reason were excluded from the final sample.

PD: Parkinson's disease; AA: African American; SUD: substance use disorder; DME: durable medical equipment; HHA: Home Health Agency; SNF: skilled nursing facility; S.D.: Standard Deviation.

^*∗∗∗*^ *p* < .001; ^*∗∗*^.001 ≤ *p* < .01; ^*∗*^.01 ≤ *p* < .05.

**Table 2:** Number and percent with home healthcare use and average home healthcare expenditures among users by Parkinson's disease status among elderly Medicare beneficiaries. National Medicare 5% sample, 2006-2007.

All	Percent with home healthcare use					Average home healthcare expenditures among users				
	PD		No PD		Sig.	PD		No PD		Sig.
	2,445	22.5	4,601	9.2	*∗∗∗*	$6,792	$6,640	$4,965	$5,537	
Predisposing characteristics										
Gender										
Female	1,348	24.9	3,148	10.5	*∗∗∗*	$7,055	$198	$5,201	$108	*∗∗∗*
Male	1,097	20.2	1,453	7.3	*∗∗∗*	$6,469	$227	$4,453	$149	*∗∗∗*
Race/ethnicity										
White	2,202	22.0	3,933	8.8	*∗∗∗*	$6,556	$144	$4,601	$86	*∗∗∗*
AA	135	31.8	447	13.7	*∗∗∗*	$8,965	$648	$6,639	$312	*∗∗∗*
Others	108	24.9	221	10.6	*∗∗∗*	$8,904	$1,268	$8,048	$726	
Age group										
65–74	679	17.3	1,417	5.5	*∗∗∗*	$6,545	$288	$4,622	$164	*∗∗∗*
75, +	1,766	25.5	3,184	13.2	*∗∗∗*	$6,887	$173	$5,117	$103	*∗∗∗*

Enabling characteristic										
Public assistance										
Yes	392	22.5	875	15.3	*∗∗∗*	$8,368	$514	$6,785	$286	*∗∗*
No	2,053	22.5	3,726	8.4	*∗∗∗*	$6,491	$142	$4,537	$84	*∗∗∗*

External environmental characteristics										
Census regions										
Northeastern	562	23.4	996	10.4	*∗∗∗*	$5,832	$261	$3,800	$157	*∗∗∗*
Midwest	529	18.6	1,010	7.8	*∗∗∗*	$5,901	$219	$4,080	$128	*∗∗∗*
South	1,031	26.2	1,999	10.3	*∗∗∗*	$7,958	$278	$6,120	$162	*∗∗∗*
West	323	19.0	596	7.4	*∗∗∗*	$6,205	$308	$4,536	$182	*∗∗∗*
Metro status										
Metro	1,986	23.5	3,584	9.5	*∗∗∗*	$6,890	$174	$4,997	$104	*∗∗∗*
No metro	459	19.0	1017	8.3	*∗∗∗*	$6,372	$270	$4,852	$150	*∗∗∗*

Personal health choice										
Substance use disorder										
Yes	126	28.6	275	14.5	*∗∗∗*	$6,385	$544	$5,190	$305	*∗*
No	2,319	22.2	4,326	9.0	*∗∗∗*	$6,814	$154	$4,950	$91	*∗∗∗*
Inpatient visit										
Yes	1,141	31.8	1,866	20.1	*∗∗∗*	$7,971	$268	$5,699	$165	*∗∗∗*
No	1,304	17.9	2,735	6.7	*∗∗∗*	$5,761	$160	$4,464	$91	*∗∗∗*
DME visit										
Yes	1,499	32.0	2,377	16.3	*∗∗∗*	$7,766	$236	$5,891	$147	*∗∗∗*
No	946	15.3	2,224	6.3	*∗∗∗*	$5,250	$143	$3,974	$78	*∗∗∗*
Hospice visit										
Yes	19	15.6	23	17.2		$6,542	$2,600	$6,286	$1,749	
No	2,426	22.6	4,578	9.2	*∗∗∗*	$6,794	$149	$4,958	$88	*∗∗∗*
SNF visit										
Yes	499	31.2	567	28.7		$8,133	$423	$6,314	$290	*∗∗∗*
No	1,946	21.0	4,034	8.4	*∗∗∗*	$6,449	$158	$4,775	$90	*∗∗∗*
Outpatient visit quintile										
0-0	313	18.3	809	6.4	*∗∗∗*	$6,818	$392	$4,991	$207	*∗∗∗*
1-1	303	20.6	668	7.7	*∗∗∗*	$7,176	$501	$5,104	$280	*∗∗∗*
2-3	514	22.3	972	8.8	*∗∗∗*	$6,791	$325	$4,942	$191	*∗∗∗*
4–6	545	24.4	860	10.2	*∗∗∗*	$6,557	$257	$4,553	$160	*∗∗∗*
7 and higher	770	24.5	1,292	14.0	*∗∗∗*	$6,798	$275	$5,167	$168	*∗∗∗*
Office visit quintile										
0–5	72	14.3	387	4.3	*∗∗∗*	$4,786	$478	$4,134	$189	
6–10	206	15.4	630	5.5	*∗∗∗*	$5,457	$343	$4,496	$170	*∗∗*
11–16	372	17.9	816	7.7	*∗∗∗*	$6,037	$308	$4,637	$172	*∗∗∗*
17–25	570	21.7	1,048	11.1	*∗∗∗*	$6,089	$246	$4,758	$146	*∗∗∗*
26 and higher	1,225	28.4	1,720	17.7	*∗∗∗*	$7,691	$275	$5,604	$177	*∗∗∗*

Need factors										
Number of comorbidities										
<=16	623	15.4	1,552	5.3	*∗∗∗*	$5,649	$195	$4,207	$104	*∗∗∗*
>16	1,822	26.8	3,049	14.8	*∗∗∗*	$7,183	$1,942	$5,350	$118	*∗∗∗*

Note: based on 2,445 and 4,601 elderly (age 65 or older) Medicare beneficiaries with and without Parkinson's disease who were continuously enrolled in Medicare Part A and Part B during 2006 and 2007 and were not enrolled in Health Maintenance Organizations during 2006 and 2007; individuals with end-stage renal disease (ESRD) as well as those without full year enrollments due to death or some other reason were excluded from the final sample. Average home healthcare expenditures were estimated among users of home healthcare.

S.E.: Standard Errors; PD: Parkinson's disease; AA: African Americans; DME: durable medical equipment; SNF: skilled nursing facility.

^*∗∗∗*^ *p* < .001; ^*∗∗*^.001 ≤ *p* < .01; ^*∗*^.01 ≤ *p* < .05.

**Table 3:** Adjusted Odds Ratios and 95% Confidence Intervals from logistic regression on home healthcare use and parameter estimates from OLS regression on log-transformed home healthcare expenditures among users. National Medicare 5% sample, 2006-2007.

	AOR	95% CI	Sig.	Beta	S.E.	Sig.
Intercept	—	—	—	7.627	0.073	*∗∗∗*
Parkinson's disease						
Yes	2.12	[1.99, 2.25]	*∗∗∗*	0.333	0.024	*∗∗∗*
No						

Predisposing characteristics						
Gender						
Female	1.30	[1.23, 1.37]	*∗∗∗*	0.123	0.024	*∗∗∗*
Male						
Race/ethnicity						
White						
AA	1.52	[1.37, 1.68]	*∗∗∗*	0.279	0.043	*∗∗∗*
Others	1.13	[0.98, 1.29]		0.170	0.057	*∗∗*
Age group						
75, +	2.11	[1.99, 2.23]	*∗∗∗*	−0.127	0.025	*∗∗∗*
65–74						

Enabling characteristics						
Public assistance						
Yes	1.17	[1.08, 1.26]	*∗∗∗*	0.128	0.032	*∗∗∗*
No						

External environmental characteristics						
Census region						
Northeastern	1.29	[1.18, 1.41]	*∗∗∗*	−0.098	0.040	*∗*
Midwest	1.03	[0.94, 1.13]		−0.156	0.040	*∗∗∗*
South	1.40	[1.29, 1.52]	*∗∗∗*	0.196	0.036	*∗∗∗*
West						
Metro status						
Metro	1.14	[1.06, 1.21]	*∗∗∗*	0.075	0.029	*∗*
No metro						

Personal health choice						
Substance use						
Yes	1.11	[0.98, 1.25]		−0.004	0.050	
No						
Outpatient visit quintile						
0-0	1.06	[0.96, 1.16]		0.120	0.040	*∗∗*
1-1	1.08	[0.99, 1.19]		0.111	0.040	*∗∗*
2-3	1.08	[0.99, 1.17]		0.057	0.034	
4–6	1.08	[1.00, 1.17]		−0.001	0.034	
7 or higher						
Office visit quintile						
0–5	0.87	[0.76, 1.00]		−0.071	0.062	
6–10	0.87	[0.78, 0.97]	*∗*	−0.021	0.048	
11–16	0.96	[0.87, 1.05]		−0.009	0.040	
17–25	1.05	[0.97, 1.14]		−0.037	0.033	
26 or higher						
SNF visits	0.88	[0.85, 0.91]	*∗∗∗*	0.052	0.012	*∗∗∗*
Inpatient visits	1.22	[1.18, 1.26]	*∗∗∗*	0.033	0.013	*∗*
Hospice visits	0.97	[0.93, 1.01]		0.001	0.016	
DME visits	1.05	[1.05, 1.06]	*∗∗∗*	0.016	0.002	*∗∗∗*

Need factors						
Number of comorbidities	1.04	[1.03, 1.04]	*∗∗∗*	0.006	0.002	*∗∗∗*

Note: logistic regression conducted on 10,865 and 50,009 elderly (age 65 or older) Medicare beneficiaries with and without Parkinson's disease who were continuously enrolled in Medicare Part A and Part B during 2006 and 2007 and were not enrolled in Health Maintenance Organizations during 2006 and 2007; they had a positive direct total healthcare expenditure; individuals with end-stage renal disease (ESRD) as well as those without full year enrollments due to death or some other reason were excluded from the final sample.

Ordinary Least Squares regression conducted on 2,445 and 4,601 elderly (age 65 or older) Medicare beneficiaries with and without Parkinson's disease who were continuously enrolled in Medicare Part A and Part B during 2006 and 2007 and were not enrolled in Health Maintenance Organizations during 2006 and 2007; they were home healthcare users; individuals with end-stage renal disease (ESRD) as well as those without full year enrollments due to death or some other reason were excluded from the final sample.

OLS: Ordinary Least Squares; S.E.: Standard Errors; PD: Parkinson's disease; AA: African Americans; SUD: substance use disorder; DME: durable medical equipment; SNF: skilled nursing facility.

^*∗∗∗*^ *p* < .001; ^*∗∗*^.001 ≤ *p* < .01; ^*∗*^.01 ≤ *p* < .05.

**Table 4:** Nonlinear and linear decomposition of home healthcare use and expenditures. Elderly Medicare beneficiaries with and without PD. Medicare 5% claims database (2006, 2007).

% with home healthcare use (PD) % with home healthcare use (no PD)	**22.5%** **9.2%**	

Average log-transformed home healthcare expenditures (PD)		**8.44**
Average log-transformed home healthcare expenditures (no PD)		**8.09**

Difference	**13.3**	**0.35**

Total explained	**3.64**	**0.063**

	Nonlinear decomposition	Linear decomposition

Predisposing characteristics		
Gender, race/ethnicity, age	5.3%	1.8%
Enabling characteristic		
Public assistance	−1.5%	1.6%
Need characteristic		
Number of comorbidities	17.5%	4.1%
Personal health choices		
Any type of SUD Baseline resource use	6.0%	12.6%
External environmental factors		
Census region Metro status	0.2%	−2.1%

Explained to total difference	27.5%	18.0%
Unexplained portion	72.5%	82.0%

Note: nonlinear decomposition analysis based on 10,865 and 50,009 elderly (age 65 or older) Medicare beneficiaries with and without PD.

Difference in home healthcare use = 13.3 percentage points. Percentage points in home healthcare use are explained by each independent variable. The percentage explained is derived by dividing the total explained portion by the 13.3-percentage-point difference between elderly Medicare beneficiaries with and without PD.

Linear decomposition analysis based on 2,445 and 4,601 elderly (age 65 or older) Medicare beneficiaries with and without PD and who used home healthcare.

SUD: substance use disorder; Exp.: expenditure.
